# From Cats to the Cortex: Unravelling the Hierarchical Processing System of Vision and Brain Plasticity

**DOI:** 10.7759/cureus.68424

**Published:** 2024-09-02

**Authors:** Mujeeb Ur Rehman Parrey

**Affiliations:** 1 Ophthalmology, Department of Surgery, Northern Border University, Arar, SAU

**Keywords:** robotics, interdisciplinary influence, artificial intelligence in medicine, ocular dominance columns, nobel prize, historical vignette, critical period of visual development, congenital cataract, architecture of visual cortex, amblyopia

## Abstract

The groundbreaking research conducted by neurophysiologists David Hubel and Torsten Wiesel during the late 1950s and 1960s revolutionized the field of visual neuroscience. Through single-unit recordings in the visual cortex of cats, they made several key discoveries that fundamentally changed our understanding of visual processing. Their work introduced the concept of orientation selectivity, revealing that neurons in the visual cortex are specifically tuned to line orientations, thereby illustrating how the brain constructs visual representations through edge detection. Additionally, they discovered ocular dominance columns, the specialized cortical regions that respond preferentially to input from one eye, providing crucial insights into the organization of visual processing and the importance of binocular vision.

Hubel and Wiesel’s research also established the concept of a critical period in visual development, demonstrating that early visual experiences are essential for the proper maturation of the visual system. This discovery has had significant implications for understanding neural plasticity and the role of sensory input in neural development. The impact of their work goes beyond theoretical knowledge, contributing to the development of therapeutic strategies for some visual disorders and guiding current research into brain plasticity and visual processing.

This review synthesizes the monumental contributions of Hubel and Wiesel, evaluating how their key discoveries have shaped subsequent research in visual neuroscience. It traces the evolution of knowledge related to visual pathways, feature detection, and brain plasticity, highlighting the enduring influence of their foundational work on contemporary studies. By exploring the progression from their pioneering findings to modern advancements, this review emphasizes the legacy of Hubel and Wiesel’s contributions to our understanding of vision and neural function.

## Introduction and background

The study of visual perception has undergone significant transformations, largely driven by the pioneering research of neurophysiologists David Hubel and Torsten Wiesel. Their collaborative efforts from the late 1950s through the 1960s greatly advanced our understanding of how the brain processes visual information. Central to their research was the investigation of the visual cortex in cats, where they used single-unit recordings to monitor the activity of individual neurons. This innovative technique paved the way for some of the most significant discoveries in visual neuroscience.

One of Hubel and Wiesel’s landmark contributions was identifying orientation selectivity in the neurons of the visual cortex. Around 1959, they discovered that specific neurons respond selectively to lines or edges of orientations, such as vertical or horizontal. This finding was crucial in explaining how the brain assembles a cohesive visual representation from various sensory inputs. In the early 1960s, Hubel and Wiesel also discovered the concept of ocular dominance columns, distinct regions within the visual cortex where neurons show a preference for input from one eye over the other. This discovery was vital in understanding the organization of visual processing and highlighted the importance of binocular vision for depth perception and spatial awareness.

Another significant breakthrough came in the mid-1960s with their identification of a critical period in visual development. Through experiments on kittens, in which one eye was sutured, they demonstrated that early visual experiences are essential for the proper development of the visual system. This research not only deepened our understanding of brain plasticity but also underscored the crucial role of sensory input in shaping neural circuits during critical developmental periods.

**Figure 1:**
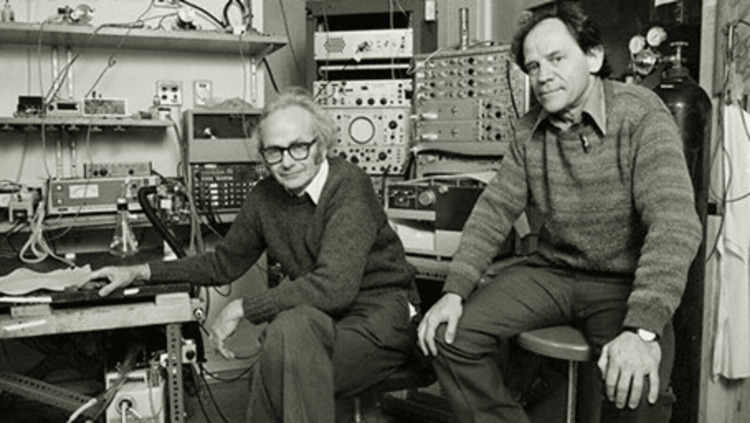
Hubel and Wiesel following their Nobel Prize win in 1981. Credit: Defining Moments Canada [1]. Source: Licensed under Attribution-NonCommercial International License CC BY-NC 4.0.

These discoveries made a lasting impact on the field of visual neuroscience, offering valuable insights into the hierarchical processing within the visual system and the influence of experience on brain development. Hubel and Wiesel’s research has continued to inspire subsequent studies, leading to further advancements in our understanding of visual pathways, feature detection, and the treatment of visual disorders. Their work was deservedly honored with the Nobel Prize in Physiology or Medicine in 1981, solidifying their legacy in neuroscience [1]. Figure 1 represents a picture of Hubel and Weisel taken after winning their Nobel Prize.

The implications of their contributions extend beyond visual perception, influencing diverse research areas such as brain plasticity and computational models of vision. Building on their foundational discoveries, subsequent research has expanded our knowledge of the visual system. Progress includes the mapping of higher-order visual pathways, exploration of binocular vision and feature detection, and the development of new technologies like non-invasive brain imaging and neural prosthetics. The field has also benefited from the contributions of other notable scientists, including Brenda Milner and Semir Zeki, who have furthered our understanding of visual processing and its underlying neural mechanisms.

## Review

This review delves into the groundbreaking research of David Hubel and Torsten Wiesel on the visual system, emphasizing their discoveries on how the brain processes visual information. Their work in the 1950s and 1960s, primarily with cats, uncovered orientation selectivity and ocular dominance columns, shedding light on critical aspects of visual perception and brain development. Through the introduction of single-unit recording techniques to study neuron activity, they significantly advanced the field of neuroscience.

The review also highlights the extensive impact of their work on brain plasticity research, the development of treatments for some visual disorders, and innovations like visual prosthetics. It acknowledges their 1981 Nobel Prize and the lasting influence of their research on modern neuroscience, particularly through comparative and interdisciplinary studies. Limitations, including species-specific findings, are also mentioned. Ultimately, the review underscores Hubel and Wiesel's legacy and their crucial role in advancing the understanding of the brain's visual processing mechanisms.

Biographical background

David Hubel (1926-2013) was born on February 27, 1926, in Windsor, Ontario, Canada. David Hunter Hubel developed a keen interest in science and medicine early in life, a passion that would shape his future career. Encouraged by his family, Hubel enthusiastically explored various scientific disciplines. After completing his undergraduate studies at McGill University, he earned a medical degree from McGill's Faculty of Medicine, where he became deeply interested in the nervous system. He then moved to the United States for further academic pursuits, completing a residency in neurophysiology at Johns Hopkins University. There, Hubel focused on studying neuronal electrical activity using early electrophysiological techniques. It was at Johns Hopkins that he met Torsten Wiesel, beginning a highly productive partnership that led to groundbreaking discoveries in neuroscience.

Torsten Wiesel (1924-2020) was born on June 3, 1924, in Uppsala, Sweden. Raised in an academic family, with his father as a physiology professor and his mother deeply interested in literature and the arts, Wiesel grew up in an intellectually stimulating environment that fostered his curiosity and passion for learning. He pursued a medical degree at the Karolinska Institute in Stockholm, focusing on neuroscience. After completing his studies, Wiesel moved to the United States for postgraduate training, eager to deepen his understanding of neurophysiology. He first worked at the University of Chicago before joining Johns Hopkins University, where he crossed paths with David Hubel. This meeting led to one of the most successful collaborations in the history of neuroscience. Wiesel's meticulous research, creativity, and intellectual rigor perfectly complemented Hubel’s analytical skills, forming a powerful partnership in the field.

Collaborations and innovations

**Figure 2:**
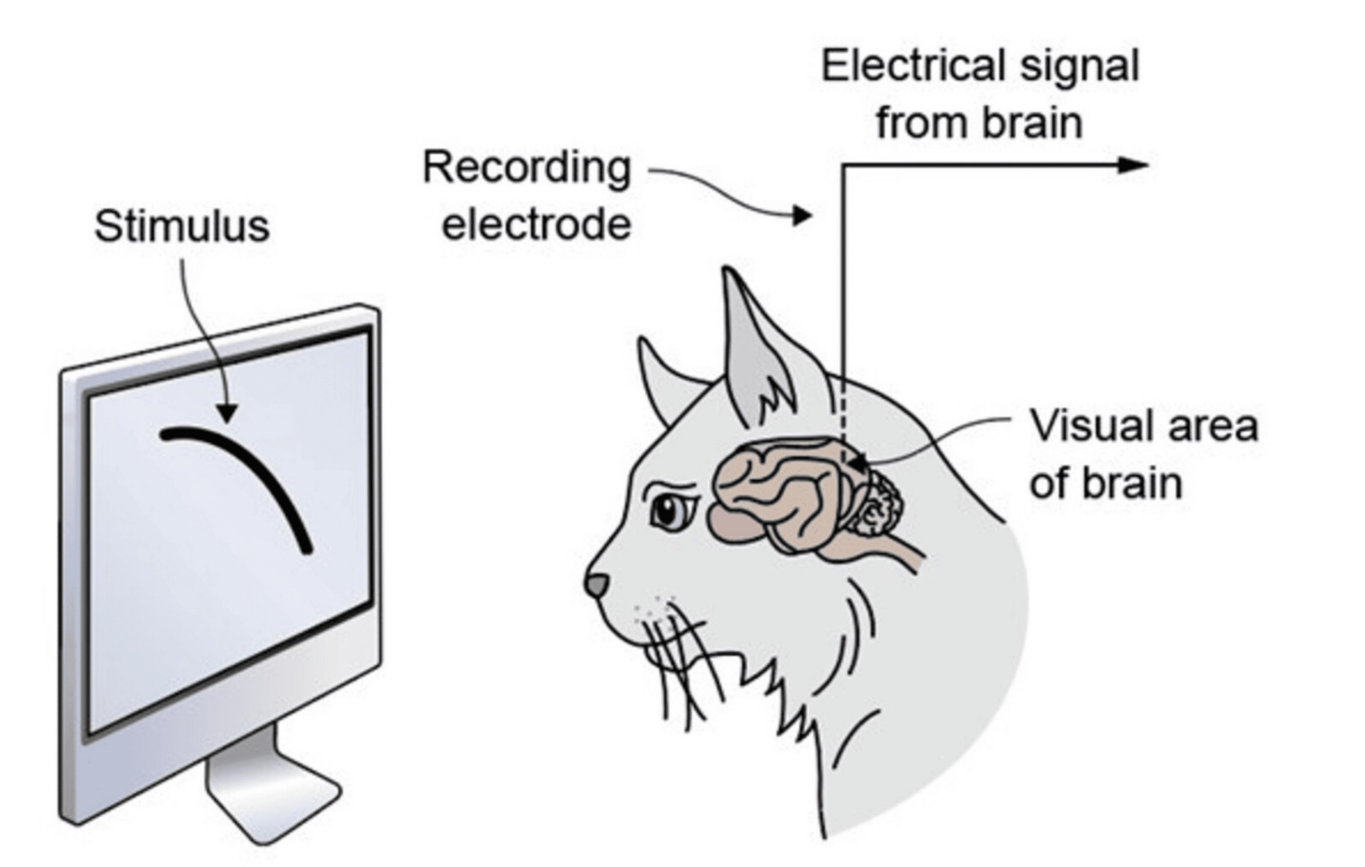
Hubel and Wiesel’s experiment. Credit: Defining Moments Canada [1]. Source: Licensed under Attribution-NonCommercial International License CC BY-NC 4.0.

David Hubel and Torsten Wiesel began their collaboration in the 1950s while working together at Johns Hopkins University. Their partnership blossomed into one of the most productive and impactful in the history of neuroscience. Initially, they ventured into unexplored terrain: the realm of visual physiology. Devoid of any preconceptions, they embarked on a journey to decipher the inner workings of the visual cortex, the brain region responsible for processing visual information. Their workplace, a dimly lit room in the basement of the Wilmer Institute, served as the backdrop for their experiments, where they devised a technique to observe brain activity in response to visual stimuli by implanting a sensitive electrode into the skulls of cats (Figure 2) [1].

Despite numerous unsuccessful attempts, they persisted in their efforts, often laboring late into the night. Their breakthrough came unexpectedly when a malfunctioning slide triggered a robust response in a brain cell, unveiling its sensitivity to specific visual cues. This pivotal moment marked the inception of their quest to unravel the complexities of visual processing. Through methodical experimentation, they unraveled the organization of the visual cortex and its pivotal role in perception. In a series of experiments on cats, they elucidated how signals from the eye are processed in the neocortex to form edge, motion, stereoscopic depth, and color detectors, which are essential components of visual perception. By depriving kittens of vision in one eye, they demonstrated that the columns in the primary visual cortex receiving input from the other eye would take over the regions that would normally receive visual input from the blocked eye. This finding has crucial implications for understanding deprivation amblyopia. Additionally, these kittens failed to develop regions receiving input from both eyes, which is necessary for binocular vision. Hubel and Wiesel’s experiments revealed that ocular dominance is established irreversibly early in childhood.

Contributions to neuroscience

One of the most significant discoveries made by Hubel and Wiesel was the existence of ocular dominance columns in the primary visual cortex. Their experiments revealed that neurons in the visual cortex are organized into distinct columns, each preferentially responsive to visual inputs from one eye. This finding provided crucial insights into the development and organization of the visual system, highlighting the importance of sensory experience in shaping neural circuits. Furthermore, Hubel and Wiesel's research uncovered the phenomenon of orientation selectivity, whereby neurons in the visual cortex exhibit preferential responses to specific orientations of visual stimuli. They showed that individual neurons are highly selective for specific orientations, forming a systematic map of orientation preferences across the visual cortex dedicated to extracting specific features from visual stimuli. This discovery revolutionized our understanding of visual processing, providing insights into the developmental aspects of vision in newborns, paving the way for advances in understanding the importance of early detection and management of conditions like childhood cataracts and deprivation amblyopia.

Functional architecture of the visual cortex

Building upon their initial discoveries, Hubel and Wiesel further elucidated the functional architecture of the visual cortex in greater detail. Through a series of elegant experiments, they delineated the hierarchical organization of visual processing, from simple features such as edges and contours to complex visual patterns and objects. Their work provided critical insights into how visual information is processed and represented in the brain, laying the foundation for subsequent studies on visual perception and cognition [2,3].

Legacy and impact

David Hubel and Torsten Wiesel were awarded the Nobel Prize for two significant contributions: their pioneering work on the development of the visual system, including the description of ocular dominance columns in the 1960s and 1970s, and their foundational research in visual neurophysiology. Their contributions have had a profound and lasting impact on the field of neuroscience. They illuminated our understanding of the brain's remarkable ability to perceive and interpret the visual world. Their methodological innovations set new standards for rigorous experimentation in neuroscience, inspiring generations of researchers to explore the mysteries of the brain [4-7]. Additionally, the idea of plasticity in the critical period of visual development revealed that early sensory experiences are essential for the proper maturation of the visual system, providing key insights into the brain's capacity to adapt and reorganize. This finding has been instrumental in advancing our understanding of how sensory experiences shape neural development and has significantly impacted the field of developmental neuroscience. The practical implications of Hubel and Wiesel's work extend to clinical applications, where their insights have revolutionized the modern-day approach of ophthalmologists toward the management of ophthalmic ailments like congenital cataracts and amblyopia [8,9].

Continuous development

While David Hubel and Torsten Wiesel are foundational figures in visual perception research, their pioneering work has been complemented and expanded upon by numerous other scientists who have made significant contributions to the field. David Marr developed influential computational models of vision, offering insights into how the brain processes visual information through hierarchical and modular approaches. Richard Gregory's research on perception and illusion highlighted the brain's role in constructing our visual experience and challenged traditional views of visual processing.

Brenda Milner's work in neuropsychology, particularly with patients with brain lesions, illuminated the roles of different brain regions in vision and memory, advancing our understanding of cognitive functions [10]. Takao Hensch’s research into the critical period for visual development furthered our knowledge of how early sensory experiences impact neural development and improved clinical treatments for visual disorders like amblyopia [8]. Semir Zeki's exploration of higher-order visual processing examined how the brain recognizes and categorizes objects, contributing to our understanding of visual areas beyond the primary cortex [11]. Nancy Kanwisher's use of functional magnetic resonance imaging (fMRI) techniques mapped specialized regions in the human visual cortex, identifying areas dedicated to object and face recognition [12].

Technological progress

The method of single-unit electrophysiological recording, which Hubel and Wiesel pioneered, remains a fundamental technique in neuroscience. It involves inserting fine electrodes into the brain to record the electrical activity of individual neurons. This technique has been refined and adapted over time, contributing to our understanding of neural circuits and sensory processing [13]. Building on the principles of single-unit recording, modern multi-electrode arrays allow simultaneous recording from multiple neurons. These arrays provide more comprehensive data on neuronal interactions and network dynamics, advancing our understanding of complex brain functions [14].

While not directly developed by Hubel and Wiesel, their research laid the groundwork for modern imaging techniques such as functional MRI (fMRI) and positron emission tomography (PET). fMRI uses blood oxygenation level-dependent (BOLD) contrast to visualize brain activity in response to various stimuli. The foundational understanding of visual processing and cortical organization from their work has been critical in developing and interpreting fMRI studies [15]. Techniques like optogenetics, which allows precise control of neural activity using light, have been developed to further explore the visual system. Although this technology emerged after Hubel and Wiesel's era, the principles of neural selectivity and function derived from their research have influenced the development of optogenetics, which is used to study and manipulate neural circuits in real time [16].

The understanding of visual processing and neural circuits has contributed to the development of visual prosthetics, such as retinal implants and other prosthetic devices. Devices like the Argus II Retinal Prosthesis System aim to restore vision in individuals with retinal degenerative diseases by directly stimulating the remaining retinal cells or the visual cortex [17]. Hubel and Wiesel's research on cortical processing and neural plasticity has informed the development of brain-computer interfaces. These devices can decode neural signals to control external devices, providing new ways to assist individuals with sensory or motor impairments [18].

Hubel and Wiesel’s research has directly informed the diagnosis and treatment of some visual disorders. The insights gained from their research have led to the development of diagnostic tools and early treatment approaches for amblyopia and pediatric cataracts to prevent permanent vision loss. Technologies such as specialized visual acuity tests and interactive vision therapy systems are designed to address visual deficiencies identified through studies of critical periods and sensory experience. Advanced therapies that target neural plasticity and critical periods are informed by Hubel and Wiesel's findings. These include visual training programs and rehabilitation technologies aimed at improving vision and neural function in individuals with visual impairments [19]. Insights into the visual processing pathways and neuroplasticity have been applied in rehabilitative medicine to help patients recover from strokes, traumatic brain injuries, and other neurological conditions affecting vision and motor coordination.

Modern electroencephalography (EEG) and magnetoencephalography (MEG) systems, which measure electrical and magnetic activity in the brain, are used to study visual processing and cortical function. These technologies benefit from the foundational knowledge provided by Hubel and Wiesel's research on cortical maps and processing [20].

Comparative analysis

To frame a comparative analysis of Hubel and Wiesel’s research, their findings can be assessed alongside other key neuroscience studies from their time and subsequent ones. Prior to Hubel and Wiesel’s work, the understanding of visual processing was relatively rudimentary. The prevailing theories were largely speculative, lacking empirical data on how the brain processes visual information at the neuronal level. Hubel and Wiesel’s research provided concrete evidence and detailed mechanisms, such as orientation selectivity and ocular dominance columns, that explained how visual stimuli were processed in the brain. This contrasted sharply with the vague and unproven theories that existed before their research. During the same period, other researchers like Stephen Kuffler were also studying the visual system, focusing on retinal ganglion cells. However, Hubel and Wiesel extended this knowledge by exploring the cortical level, showing how complex visual information is interpreted by the brain. While other researchers were making advances in sensory systems, Hubel and Wiesel’s use of single-unit recording was revolutionary, allowing them to observe individual neurons’ responses to visual stimuli. This sets a new standard for precision in neuroscience research.

Following their discoveries, the field of visual neuroscience expanded rapidly. Researchers built upon their work by exploring higher-order visual processing, such as object recognition and motion detection. Compared to later studies, Hubel and Wiesel’s work laid the foundational understanding of the primary visual cortex, which was crucial for subsequent explorations into more complex aspects of vision, such as how the brain interprets motion, color, and depth. Their findings on the importance of early sensory experiences in visual development have been echoed and expanded upon in later research on brain plasticity, showing the brain's ability to adapt to changes in sensory input over time. Compared to their contemporaries, Hubel and Wiesel’s work has had a broader interdisciplinary impact, influencing fields like artificial intelligence (AI) and machine learning, particularly in the development of neural networks that mimic the brain’s processing of visual information [21].

Enhanced interdisciplinary influence of Hubel and Wiesel’s research

Hubel and Wiesel’s pioneering work in understanding the visual cortex has transcended the boundaries of neuroscience, significantly influencing other disciplines, particularly artificial intelligence (AI) and machine learning (ML). Their discoveries have provided a biological blueprint that has inspired and shaped the development of computational models and technologies aimed at mimicking the brain’s visual processing capabilities.

One of the most notable interdisciplinary impacts of Hubel and Wiesel’s research is its influence on the development of artificial neural networks, a cornerstone of modern AI. Their work on the hierarchical processing of visual information, where simple cells in the visual cortex detect basic features like edges and orientations, and complex cells integrate these features to perceive shapes and patterns, directly inspired the architecture of convolutional neural networks (CNNs). CNNs, which are now fundamental in image recognition and computer vision, mimic this hierarchical processing by using layers of artificial neurons that progressively extract features from visual data [22]. At the time of Hubel and Wiesel’s discoveries, the field of AI was in its infancy, with early researchers exploring how machines could simulate human thought processes. Their biological findings provided a concrete model of how the brain processes information, offering AI researchers a template for developing algorithms that could replicate human-like visual recognition. Today, CNNs are employed in a wide range of applications, from facial recognition systems to autonomous vehicles and even in medical imaging for disease detection. The success of these technologies can be traced back to the principles of visual processing that Hubel and Wiesel uncovered. Their work highlighted the importance of feature extraction and hierarchical data processing, concepts that are now integral to the design of AI systems.

Beyond AI, Hubel and Wiesel’s research has influenced cognitive science, particularly in understanding perception and consciousness. Their findings on how the brain interprets sensory data have informed theories of how humans perceive the world, contributing to models of consciousness that incorporate sensory processing as a foundational element. Cognitive scientists have used their discoveries to build models that explain how the brain constructs a coherent representation of the world from fragmented sensory inputs. These models have been crucial in understanding not only normal perception but also disorders of perception, such as those seen in neurological conditions like agnosia or autism spectrum disorders.

In robotics, Hubel and Wiesel’s insights have been applied to develop more sophisticated sensory systems that allow robots to interact with their environment more effectively. By simulating the brain’s ability to process and integrate sensory information, researchers have been able to create robots with advanced visual systems that can recognize objects, navigate complex environments, and even learn from their surroundings in a manner analogous to human sensory learning. Robots used in search and rescue missions, manufacturing, and even space exploration rely on visual processing systems that can adapt to new and unpredictable environments, a capability inspired by the plasticity of the visual cortex as discovered by Hubel and Wiesel.

Finally, their research has also influenced educational tools and methods used to teach neuroscience and AI concepts. Visual processing models based on their work are now standard components of neuroscience curricula, helping students understand how complex brain functions can be broken down into simpler, more understandable processes. Additionally, public science communication often references their work to explain how the brain interprets visual information, making complex neuroscience accessible to a broader audience.

Limitations in Hubel and Wiesel’s research

While foundational for understanding visual brain function, Hubel and Wiesel's studies have some limitations. Their research primarily used cats, making it challenging to generalize the findings to humans due to differences in neural organization and visual processing. The experiments often employed simple geometric shapes and lines, which do not fully capture the complexity of real-world visual stimuli.

Their focus on the critical period of visual development highlighted the importance of early experiences but mainly addressed the effects of deprivation, without considering other forms of sensory input or atypical development beyond these critical periods. Additionally, the single unit recording technique they used, although innovative, only provided insights into individual neurons, missing the broader context of neural network interactions. Furthermore, their work predominantly investigated static visual stimuli, leaving gaps in understanding dynamic visual processing, such as motion perception and the integration of visual information over time.

## Conclusions

David Hubel and Torsten Wiesel's collaboration represents a paradigmatic example of scientific excellence and discovery. Their pioneering research on the neural basis of vision revolutionized the field of neuroscience, shaping our understanding of brain function and perception. Through their innovative experiments and insightful observations, they uncovered fundamental principles of neural processing that continue to inspire and inform research in neuroscience to this day. In recognition of their pioneering work, Hubel and Wiesel were jointly awarded the Nobel Prize in Physiology or Medicine in 1981, cementing their status as giants in the field of neuroscience.

Their legacy stands as a testament to the power of curiosity, collaboration, and perseverance in advancing our understanding of the complexities of the human brain. Their influence endures through the many scientists inspired by their work and the lasting impact of their ideas on our comprehension of the brain and its remarkable capabilities.
